# Automated continuous noninvasive ward monitoring: future directions and challenges

**DOI:** 10.1186/s13054-019-2485-7

**Published:** 2019-05-30

**Authors:** Ashish K. Khanna, Phillip Hoppe, Bernd Saugel

**Affiliations:** 1 Department of Anesthesiology, Section on Critical Care Medicine, Wake Forest University School of Medicine, Wake Forest Baptist Health, Winston-Salem, NC USA; 2 Outcomes Research Consortium, Cleveland, OH USA; 3 Department of Anesthesiology, Center of Anesthesiology and Intensive Care Medicine, University Medical Center Hamburg-Eppendorf, Martinistrasse 52, 20246 Hamburg, Germany

**Keywords:** Hemodynamic monitoring, Postoperative complications, Blood pressure, Hypotension, Peripheral oxygen saturation, Hypoxemia, Remote monitoring, Normal ward, Artifacts, False alarms

## Abstract

Automated continuous noninvasive ward monitoring may enable subtle changes in vital signs to be recognized. There is already some evidence that automated ward monitoring can improve patient outcome. Before automated continuous noninvasive ward monitoring can be implemented in clinical routine, several challenges and problems need to be considered and resolved; these include the meticulous validation of the monitoring systems with regard to their measurement performance, minimization of artifacts and false alarms, integration and combined analysis of massive amounts of data including various vital signs, and technical problems regarding the connectivity of the systems.

Patient monitoring by definition is the repeated or continuous observation of vital signs or physiologic functions to ensure patient safety and guide therapeutic interventions. Today, most advanced cardiorespiratory monitoring systems depend on invasive sensors, cables, and bulky monitors to recognize, transfer, process, and display the bio-signals to be monitored. Therefore, continuous advanced cardiorespiratory monitoring is mainly restricted to the intensive care unit, the operating room, and the post anesthesia care unit. Most other monitoring in the hospital continues to be basic and intermittent—including monitoring on medical and surgical general care wards. When at home, before-and-after hospital admission, patients are usually not monitored at all [1].

While most advanced monitoring is in place in intensive care units, nearly half of all adverse events in hospitalized patients occur on the general care ward [2–4]. Ironically, this area—also referred to as “the patient’s room”—is traditionally regarded as a place of recovery for the more stable medical or surgical patients, who will (in the absence of setbacks) transition to leave the hospital. In addition, the European Surgical Outcomes Study (EuSOS) [5] revealed that about three quarters of patients who died in the hospital after surgery were not admitted to an intensive care unit at any stage after surgery; this indicates that the general care ward plays a pivotal role in the care for patients in the postoperative period, a period in which patients are especially prone to developing clinical deterioration and life-threatening complications [5, 6]. Not only are catastrophic cardiorespiratory events common in general care ward environments, their outcomes are significantly worse compared with similar events in monitored intensive care units. For example, a large national registry identified 44,551 index events across more than 300 US hospitals [7]. More importantly these acute respiratory events on inpatient wards had an associated in-hospital mortality of approximately 40% [7].

Current ward monitoring protocols typically consist of intermittent spot checks by a nurse about every 4–8 h. This leaves patients unmonitored for most of the time during their hospital stay [8]. Alterations in vital signs as warning signs of clinical deterioration are frequently not or only belatedly recognized by the conventional spot check-based monitoring strategy. In hospitalized patients recovering from non-cardiac surgery, severe prolonged hypoxemia is common and unfortunately seriously underestimated using intermittent vital sign checks [9, 10]. Similar patterns regarding the rate of recognized abnormal vital signs were observed for tachycardia, bradycardia, tachypnea, and bradypnea [10]. In patients recovering from abdominal surgery on the general care ward, postoperative hypotension (mean arterial pressure < 65 mmHg for ≥ 15 min) has been shown to occur in about one fifth of patients and not to be recognized by routine vital sign assessments in about half of the cases [11]. In addition to missing critical changes in vital signs, the recognition of abnormal vital signs by a bedside nurse often triggers a long chain of commands resulting in delays until an intervention can be taken [12].

A closed claims analysis of opioid induced respiratory compromise on the general care ward identified nearly half of all these events occurred within 2 h of the last nursing check [13]. In addition, the authors concluded that nearly all of these events would have been prevented by better continuous monitoring and education [13]. Considering that most nursing spot checks of vital signs leave gaps of about 4 h in-between two consecutive assessments, this period is associated with the highest vulnerability.

Automated continuous noninvasive ward monitoring is a promising approach to closely follow changes in vital signs over time and thus identify patients who are deteriorating in a timely fashion (Fig. 1). The rationale behind continuous ward monitoring is that most hospitalized patients do not deteriorate all of a sudden. Although complications often become clinically apparent as acute cardiocirculatory or respiratory failure and acute changes in consciousness, we know for a long time that subtle abnormalities in vital signs usually precede these life-threatening conditions, sometimes by 6–12 h [12, 14–16]. Subtle changes in blood pressure, heart rate, respiratory rate, or oxygen saturation are early signs of clinical deterioration eventually leading to adverse events [12, 16]. Automated continuous noninvasive ward monitoring may enable a patient’s clinical deterioration to be identified well before a serious adverse event occurs [16]. Further, novel monitoring technologies also may enable advanced hemodynamic variables such as stroke volume, cardiac output, and dynamic cardiac preload parameters to be monitored continuously in patients on the general care ward [17–19]; to date, these variables—in contrast to surgical or critically ill patients—play no role in the treatment of patients in this environment.

**Fig. 1:**
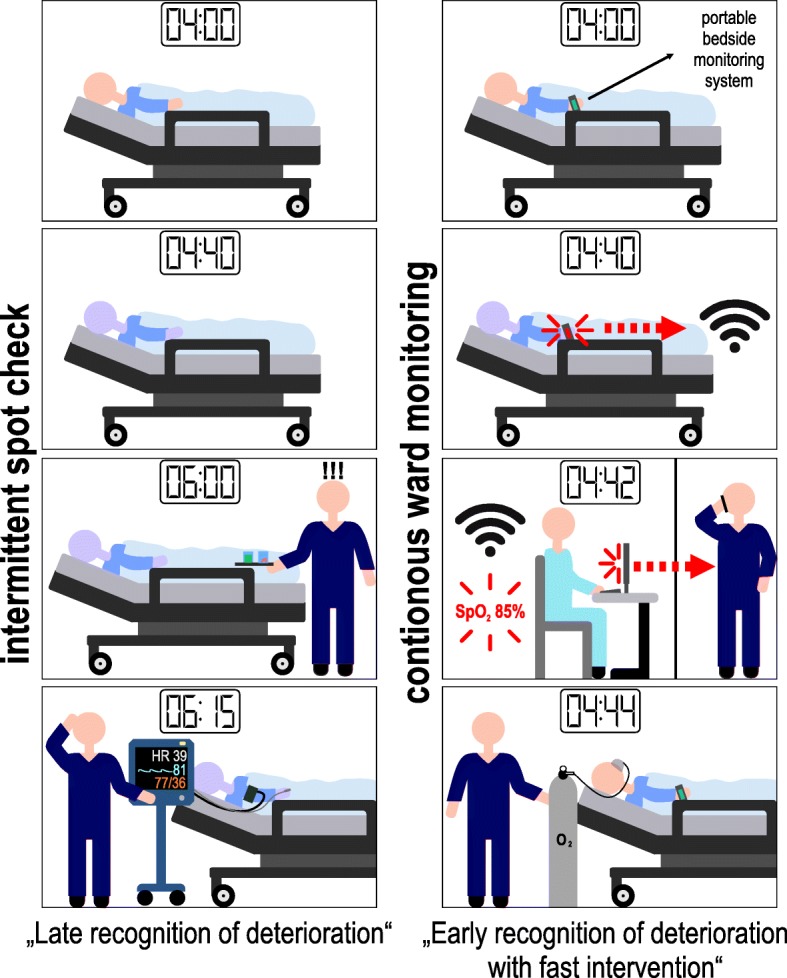
Automated continuous noninvasive ward monitoring allows the healthcare provider to closely follow changes in vital signs over time and identify patients who are deteriorating earlier than conventional intermittent spot check monitoring. Early recognition of clinical deterioration enables rapid therapeutic interventions which may be life saving in certain situations

There is already some evidence that intensified and automated ward monitoring of vital signs may improve patient outcome by a reduction of rescue events [1]. Before-and-after studies showed that the deployment of an electronic automated advisory vital signs monitoring and notification system is associated with significant improvements in key patient-centered clinical outcomes in patients treated on the normal ward [20, 21]. In an orthopedic ward, the implementation of a continuous pulse oximetry surveillance system linked to a nursing notification system reduced the number of rescue events from 3.4 to 1.2 per 1000 patient discharges and also reduced the rate of intensive care unit transfers (before-and-after study) [22]. In another study, the implementation of a system allowing for continuous monitoring of heart and respiratory rate in a medical-surgical unit was associated with lower “code blue” rates (6.3 before to 0.9 after implementation per 1000 patients) [23].

Although continuous ward monitoring is not standard of care today, innovative monitoring systems—in theory—would already allow us to noninvasively and continuously monitor heart rate, blood pressure, respiratory rate, oxygen saturation, skin temperature, body posture, activity, and location within the hospital [24–28]. Battery powered, wearable or adhesive, wireless monitoring systems that communicate with mobile devices or patient monitors may—in the near future—give hospitalized patients the freedom to move within their rooms and the hospital while being monitored [25, 28]. While, the best-case scenario would be the implementation of universal continuous smart monitoring for all inpatients, there may also be value in attempting to identify the highest risk strata of those most likely to face sudden unprecedented episodes of cardiorespiratory compromise. Novel scores such as PRODIGY developed using continuous capnography and oximetry may help the perioperative clinician in early interventions using a combination of better monitoring and other proactive strategies to avert future problems [29].

Furthermore, before automated continuous noninvasive ward monitoring becomes a reality in routine clinical care outside of studies, several problems and limitations need to be considered. Most importantly, monitoring systems need to be reliable, accurate, and be able to provide readings of vital signs with a low rate of artifacts and false alarms. However, some of the currently available monitoring systems lack clinically acceptable accuracy and precision [30]. Therefore, meticulous validation needs to precede the use of novel cardiorespiratory monitoring systems in studies or clinical practice. Especially for blood pressure, a key hemodynamic variable, reliable continuous noninvasive monitoring is technically challenging and unavailable in most smart portable systems [27]. Some monitoring systems still suffer from high rates of artifactual readings and false alarms [31]. Not only is the problem of false alarms a nuisance, it will almost always lead to an increasing level of alarm fatigue within bedside providers, the so called first-responders to needless alarms. In this regard, some vital signs are more prone to artifacts and false alarms than others—for example, capnography as a measure of ventilation being one that has always been a prime suspect for this. Frequent and false alarms may be automatically identified and reduced by cross-checking and machine learning algorithms [32–34].

Automated continuous noninvasive ward monitoring of a variety of vital signs with one or more sensors will create a massive amount of data that need to be processed in real-time, stored, and secured. These data reflecting different bio-signals will need to be integrated and analyzed together to allow the identification of certain patterns of vital sign alterations instead of merely recognizing that single values of single variables are outside of their normal range. Several predictive statistical models have already been developed, validated, and embedded in electronic medical records as automated aggregated “early warning scores” that assign weights to altered vital signs proportionate to their deviation from normal ranges [35–37]. Identifying changes in physiologic variables over time and using machine and deep learning methods may improve the predictive capabilities of these risk stratification tools [38, 39].

Other challenges concern the technical connectivity between sensors and monitoring systems. While the “internet of things” (i.e., a network of devices, vehicles, and home appliances) became part of our daily life, wireless data transmission and processing are not yet well established in hospitals and other health care facilities. Problems for the implementation of wireless monitoring systems include—but are not limited to—range, power consumption, integration in electronic health records, and cybersecurity [1]. Legal issues regarding data protection and privacy rights are beyond the scope of this article but are essential topics that need to be taken care of before ward monitoring can be implemented in healthcare systems.

Finally, before automated continuous noninvasive ward monitoring can be recommended for routine clinical use, we need to await the results of adequately powered randomized controlled trials demonstrating its effectiveness in improving the quality of care and carefully chosen patient-centered outcomes. As a next step, research may then focus on investigating which patients benefit from expanding ward monitoring to home monitoring in the period after hospital discharge [1, 40].

## Conclusions

Automated continuous noninvasive ward monitoring seems to be an intriguing opportunity to timely detect clinical problems by recognizing subtle changes in vital signs and improve patient outcomes on the general care ward. There is already some evidence—mainly from before-and-after studies—that automated ward monitoring can improve patient outcome. From a technical point of view, monitoring systems for automated continuous noninvasive ward monitoring are already available and will be further refined during the next years, probably resulting in small, wireless, and wearable sensors. However, before automated continuous noninvasive ward monitoring can be implemented in clinical routine, several challenges and problems need to be considered and resolved; these include the meticulous validation of the monitoring systems with regard to their measurement performance, minimization of artifacts and false alarms, integration and combined analysis of massive amounts of data including various vital signs, and technical problems regarding the connectivity of the systems. The primary scientific aim, though fairly simple, needs some thought and well-planned trial design, and would look to evaluate in robust and adequately powered trials whether automated continuous noninvasive ward monitoring can improve patient outcome compared with current standard spot-check monitoring. Till such time, it seems rather inappropriate to leave our patients under-monitored and unprotected for large periods of time as they recover from illness on our general care hospital wards.

## Data Availability

Not applicable
